# Treatment patterns and survival after 18F-fluorodeoxyglucose positron emission tomography/computed tomography-guided local consolidation therapy for oligometastatic non-small cell lung cancer: a two-center propensity score-matched analysis

**DOI:** 10.1007/s00432-020-03134-9

**Published:** 2020-01-25

**Authors:** Ying-Qiu Song, Nan Wang, Yun Qiao, Lei He, Xia Li, Xiao-Fang Zhang, Qian-Kun Yang, Run-Ze Wang, Rong He, Chen-Yu Wang, Yang-Wu Ren, Guang Li, Tian-Lu Wang

**Affiliations:** 1 Department of Radiotherapy, Cancer Hospital of China Medical University, Liaoning Cancer Hospital and Institute, Shenyang, Liaoning China; 2 Department of Surgery, Cancer Hospital of China Medical University, Liaoning Cancer Hospital and Institute, Shenyang, Liaoning China; 3 Physical Laboratory in Charge, Department of Radiotherapy Department, Liaoning Cancer Hospital and Institute, Shenyang, Liaoning China; 4 China Medical University, Shenyang, Liaoning China; 5 Chengdu Medical College, Chengdu, Sichuan China; 6 Department of Cerebral Surgery, Liaoning Cancer Hospital and Institute, Shenyang, Liaoning China; 7 Department of Information Management, Liaoning Cancer Hospital and Institute, Shenyang, Liaoning China; 8 Department of Epidemiology, School of Public Health, China Medical University, Shenyang, Liaoning China; 9 Department of Radiotherapy, The First Hospital of China Medical University, Shenyang, Liaoning China

**Keywords:** Non-small cell lung cancer, Oligometastasis, Local consolidation therapy, Prognosis

## Abstract

**Purpose:**

In this retrospective study, we evaluated the treatment patterns and survival after positron emission tomography-computed tomography (PET/CT)-guided local consolidation therapy (LCT) for oligometastatic non-small cell lung cancer (NSCLC).

**Methods:**

We reviewed the medical records of Chinese patients with oligometastatic stage IV non-small cell lung cancer (≤ 5 metastases) who had undergone PET/CT and were eligible for systemic therapy at two centers between May 2005 and August 2019. Propensity score matching (1:1) was used to reduce selection bias and imbalanced distribution of confounding factors.

**Results:**

We identified 84 eligible patients and used propensity scores to create well-matched groups of 35 patients who did or did not undergo LCT. Among all patients, the 1-year overall survival (OS) rate was 47.6% and the 2-year OS rate was 22.6%. Relative to the group that did not receive LCT, the LCT group had a significantly higher OS rate (13 months vs. 7 months, *p *= 0.002). The two groups had similar incidences and classifications of LCT-related side effects. In multivariable analysis, LCT was found to be strongly associated with a favorable OS (hazard ratio: 0.508, 95% confidence interval: 0.311–0.828, *p *= 0.001).

**Conclusion:**

We concluded that LCT was significantly associated with improved clinical outcomes among the Chinese patients with oligometastatic NSCLC who were eligible for systemic treatment and could undergo PET/CT evaluation.

## Introduction

There is increasing awareness regarding the concept of oligometastasis, which, relative to extensive metastasis, is thought to involve a milder stage of tumor invasion, with fewer metastases (≤ 3–5 metastases) and localization (Ashworth et al. 2013; De Rose et al. 2016; Mitchell et al. 2019). Multiple studies have shown that patients with stage IV non-small cell lung cancer (NSCLC) generally experience progression of advanced disease at the original sites of gross disease and that patients with oligometastasis may potentially be cured via local treatment (Iyengar et al. 2018). Thus, local consolidation therapy (LCT) involving surgery and radiotherapy (RT) is expected to prolong the survival of patients with oligometastatic NSCLC.

Effective management of patients with oligometastatic lung cancer relies on accurate information regarding tumor size, location, nodal involvement, and distant disease extent, which can be obtained via imaging modalities and tissue sampling. For example, 18F-fluorodeoxyglucose positron emission tomography/computed tomography (FDG-PET/CT) has been recommended for the initial evaluation of all patients with NSCLC, as it is more likely than other staging modalities to detect advanced disease and prevent futile surgery, RT, and/or chemotherapy from being carried out. In addition, FDG-PET can more accurately identify gross tumor deposits in three-dimensional space (Wahl et al. 2011), which provides useful information for RT planning (Bradley et al. 2012; Geiger et al. 2014; Houshmand et al. 2015; Simone et al. 2016), prognostication, and treatment response monitoring in patients with NSCLC (Khiewvan et al. 2016). However, there are insufficient clinical data regarding whether LCT and/or PET/CT have a role in the management of oligometastatic NSCLC, as the existing studies have generally been single-center retrospective studies with small samples, heterogeneous disease stages, and treatments based on conventional imaging. We believe that PET/CT can help characterize a tumor’s biology and potentially help identify resistance to specific treatments, which would facilitate more effective clinical decision making regarding combined and intensified treatments. Therefore, we retrospectively evaluated real-world LCT patterns and outcomes after PET/CT-guided management of patients with oligometastatic stage IV NSCLC on the basis of data from 84 patients who were treated at two Chinese centers.

## Methods

### Patients and data sources

We retrospectively identified 289 patients with stage IV NSCLC who had undergone systemic therapy (with or without surgery or RT as LCT) at the Liaoning Cancer Hospital and the First Hospital of China Medical University between May 2005 and August 2019. Their electronic medical records were reviewed to collect information regarding age, sex, smoking history, tumor size, histological type, performance status, primary tumor sites, comorbidities, therapeutic regimens, and date of death. The inclusion criteria were stage IV NSCLC with 1–5 concurrent metastases involving the liver, brain, lungs, bones, or other locations, as well as complete records regarding surgery, RT, and/or chemotherapy. Patients at these centers should have undergone multidisciplinary consultation for surgical indications. In addition, eligible patients were required to have undergone a PET/CT examination within 1 month before treatment, on the basis of previously reported PET/CT methodology (Wang et al. 2011). Patients were excluded if they had organ dysfunction (e.g., involving the liver, kidneys, or heart), more than one primary tumor, or an unknown metastasis status, had undergone multiple transfers, or had received targeted therapy or immunotherapy.

Adverse events were evaluated according to the Common Terminology Criteria for Adverse Events (version 4.0). Patients were grouped according to whether they had received or not received LCT within 2 months before or after the administration of systemic treatment. The study’s retrospective protocol was approved by the ethics committees of the Liaoning Cancer Hospital and the First Hospital of China Medical University, and the study was conducted in compliance with the Declaration of Helsinki. All data were anonymized, and the requirement for informed consent was waived.

### Propensity score matching

Propensity score matching (PSM) was used to create groups of patients that did and did not receive LCT to reduce the influence of selection bias and confounding variables. Propensity scores were estimated using the PSM function of IBM SPSS software, and PSM was performed using 1:1 nearest neighbor matching with a caliper width of 0.02. The Chi squared test was used to examine covariate balances before and after PSM for the groups of patients with and without LCT as well as for the various subgroups.

### Statistical analysis

Demographic characteristics and clinicopathological features were reported as number (percentage) and compared using the Chi squared test. Overall survival (OS) was calculated from the date of first diagnosis to the date of death or last follow-up for surviving patients. The OS curves were estimated using the Kaplan–Meier method and compared using the log-rank test. The Cox proportional hazards model was used for univariate and multivariate analyses and the results were reported as hazard ratios (HRs) and 95% confidence intervals (CIs). Variables with *p* values < 0.1 in the univariable analysis were selected for multivariable analysis. Statistical analysis was performed using IBM SPSS software (version 25.0; IBM Corporation, Armonk, NY), and subgroup analyses according to the baseline characteristics were performed by drawing forest plots for overall survival using Stata MP 14 software (Stata Corp LLC, College Station, TX). All tests were two sided, and the results were considered significant at *p* values < 0.05.

## Results

### Follow-up and outcomes

**Fig. 1:**
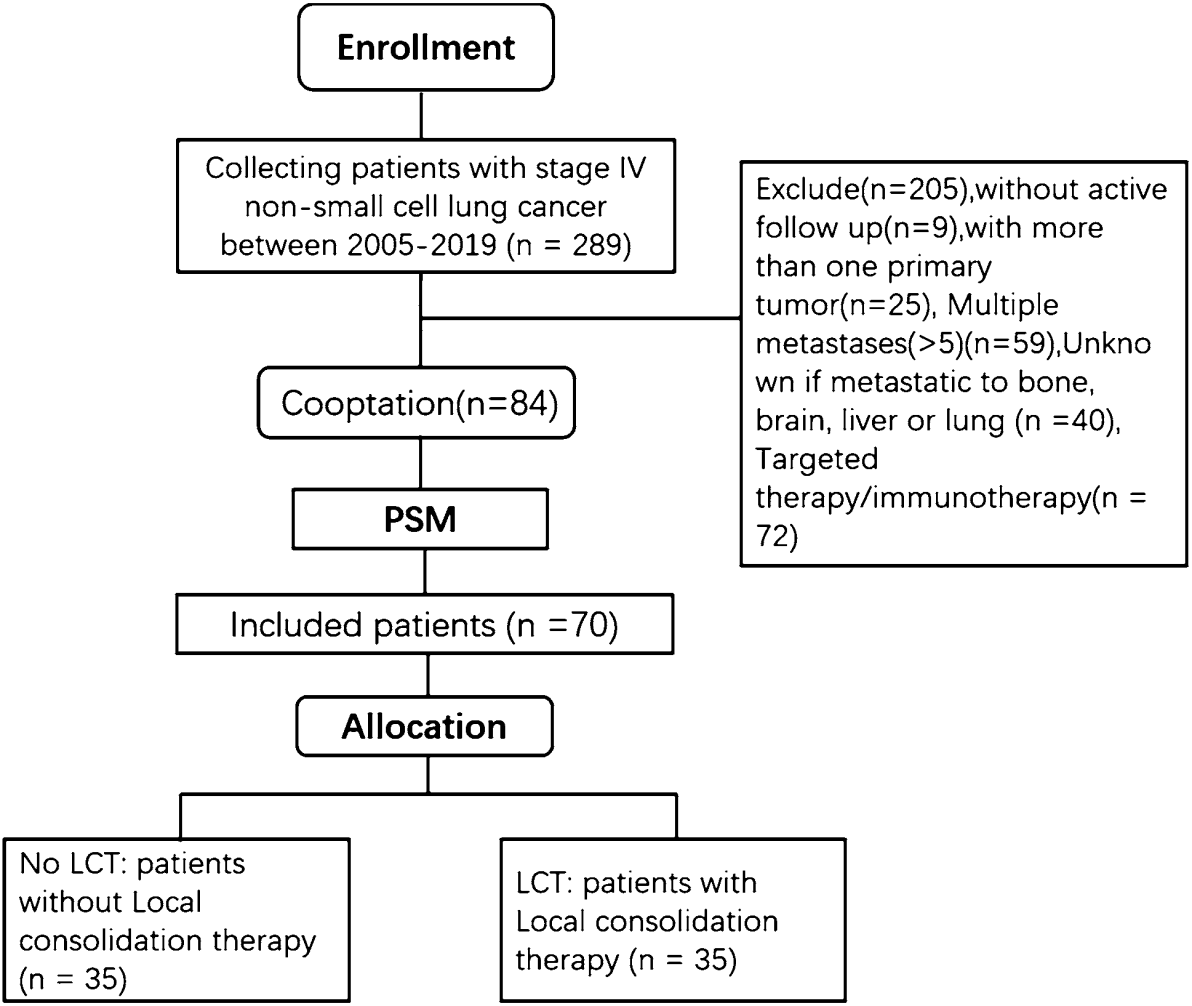
Flowchart depicting selection of the study population

Among the 289 patients treated during the study period, 205 were excluded because of targeted therapy/immunotherapy, loss to follow-up, incomplete data, or unclear metastasis status. Thus, 84 patients were considered eligible for the study. During a median follow-up of 11 months, 7 patients survived (8.3%) and 77 patients died (91.7%), with the deaths being caused by tumor-related complications (73 cases) and non-tumor-related complications (myocardial infarction in 2 cases and a cerebrovascular event in 2 cases). On the basis of the PSM, we created well-balanced groups of 35 patients who did or did not receive LCT (Fig. 1).

### Patient characteristics and distant metastasis

The 70 matched patients (35 patients in the LCT group and 35 patients in the control group) included 50 men (71.4%) and 20 women (28.6%) with pathological diagnoses of squamous cell carcinoma (28 cases, 40%), adenocarcinoma (31 cases, 44.3%), and other carcinoma types (11 cases, 15.7%). Table 1 shows the patients’ characteristics. Three patients received surgery as local treatment, 42 patients received RT (biological equivalent dose of ≥ 36 Gy), and 1 patient received surgery plus RT. There were 46 patients (65.7%) with 2–5 metastases. The most common sites were intrathoracic metastasis (41 cases, 58.6%), bone metastasis (33 cases, 47.1%), liver metastasis (15 cases, 21.4%), adrenal metastasis (8 cases, 11.4%), and brain metastasis (3 cases, 4.3%). Single-organ metastasis was observed in 33 cases, and metastasis to 2–3 organs was observed in 37 cases. The overall median survival time was 11 months (1-year OS rate: 47.6%, 2-year OS rate: 22.6%).

**Table 1:** Baseline characteristics of patients

Characteristics	Before PSM			After PSM^a^		
	NO LCT (*N* = 37) (%)	LCT (*N* = 47) (%)	*p* value	NO LCT (*n* = 35) (%)	LCT (*n* = 35) (%)	*p* value
Age (years)						
< 60	16 (43)	21 (45)	0.895	15 (43)	15 (43)	1
≥ 60	21 (57)	26 (55)		20 (57)	20 (57)	
Gender						
Male	26 (70)	31 (66)	0.674	25 (71)	25 (71)	1
Female	11 (30)	16 (34)		10 (29)	10 (29)	
Smoking						
No	12 (32)	17 (36)	0.721	10 (29)	10 (29)	1
Yes	25 (68)	30 (64)		25 (71)	25 (71)	
KPS						
> 80	30 (81)	41 (87)	0.439	28 (80)	32 (91)	0.172
≤ 80	7 (19)	6 (13)		7 (20)	3 (9)	
Pathological pattern						
SQC	11 (30)	22 (47)	0.168	11 (31)	17 (49)	0.228
AD	20 (54)	16 (34)		19 (54)	12 (34)	
Other	6 (16)	9 (19)		5 (14)	6 (17)	
Position						
Central	28 (76)	26 (55)	0.053	26 (74)	18 (51)	0.048
Peripheral	9 (24)	21 (45)		9 (26)	17 (49)	
Weight loss						
< 5%	21 (57)	26 (55)	0.895	19 (54)	21 (60)	0.629
≥ 5%	16 (43)	21 (45)		16 (46)	14 (40)	
T classification						
T1	0 (0)	1 (2)	0.014	0 (0)	1 (3)	0.082
T2	14 (38)	9 (19)		12 (34)	8 (23)	
T3	16 (43)	13 (28)		16 (46)	10 (29)	
T4	7 (19)	24 (51)		7 (20)	16 (46)	
N classification						
N0–1	7 (19)	13 (28)	0.350	7 (20)	8 (23)	0.771
N2–3	30 (81)	34 (72)		28 (80)	27 (77)	
Organ metastasis status						
Single organ	15 (41)	26 (55)	0.179	13 (37)	20 (57)	0.094
2 to 3 organs	22 (59)	21 (45)		22 (63)	15 (43)	
No. of metastasis						
Single	11 (30)	22 (47)	0.112	9 (26)	16 (46)	0.081
Multiple	26 (70)	25 (53)		26 (74)	19 (54)	
Stages of thoracic disease						
I/II	5 (14)	3 (6)	0.296	5 (14)	2 (6)	0.310
III	32 (86)	44 (94)		30 (86)	33 (94)	
Number of metastasis						
1	10 (27)	22 (47)	0.271	8 (23)	16 (46)	0.140
2	14 (38)	11 (23)		14 (40)	10 (29)	
3	5 (14)	6 (13)		5 (14)	3 (9)	
4	3 (8)	1 (2)		3 (9)	0 (0)	
5	5 (14)	7 (15)		5 (14)	6 (17)	
SUV max						
< 15	31 (84)	32 (68)	0.148	29 (83)	23 (66)	0.101
≥ 15	6 (16)	15 (32)		6 (17)	12 (34)	
Lung metastasis (yes, %)	22 (59)	28(60)	0.991	21(60)	20 (57)	0.808
Brain metastasis (yes, %)	2 (5)	3 (6)	0.851	2 (6)	1 (3)	0.555
Bone metastasis (yes, %)	20 (54)	17 (36)	0.101	19 (54)	14 (40)	0.231
Liver metastasis (yes, %)	5 (14)	11 (23)	0.252	5 (14)	10 (29)	0.145
Adrenal metastasis (yes, %)	5 (14)	6 (13)	0.92	5 (14)	3 (9)	0.452
Other metastasis (yes, %)	6 (16)	5 (11)	0.452	6 (17)	3 (9)	0.284
Mixed metastasis (yes, %)	20 (54)	18 (38)	0.150	20 (57)	13 (37)	0.094

*n* number of cases/controls, *PSM* propensity score matching, *LCT* local consolidation therapy, *SQC* squamous cell carcinoma, *AD* adenocarcinoma, *Other* other specified carcinoma

^a^The PSM was performed using age and sex, which were subdivided according to the median values

### Prognostic factors

**Fig. 2:**
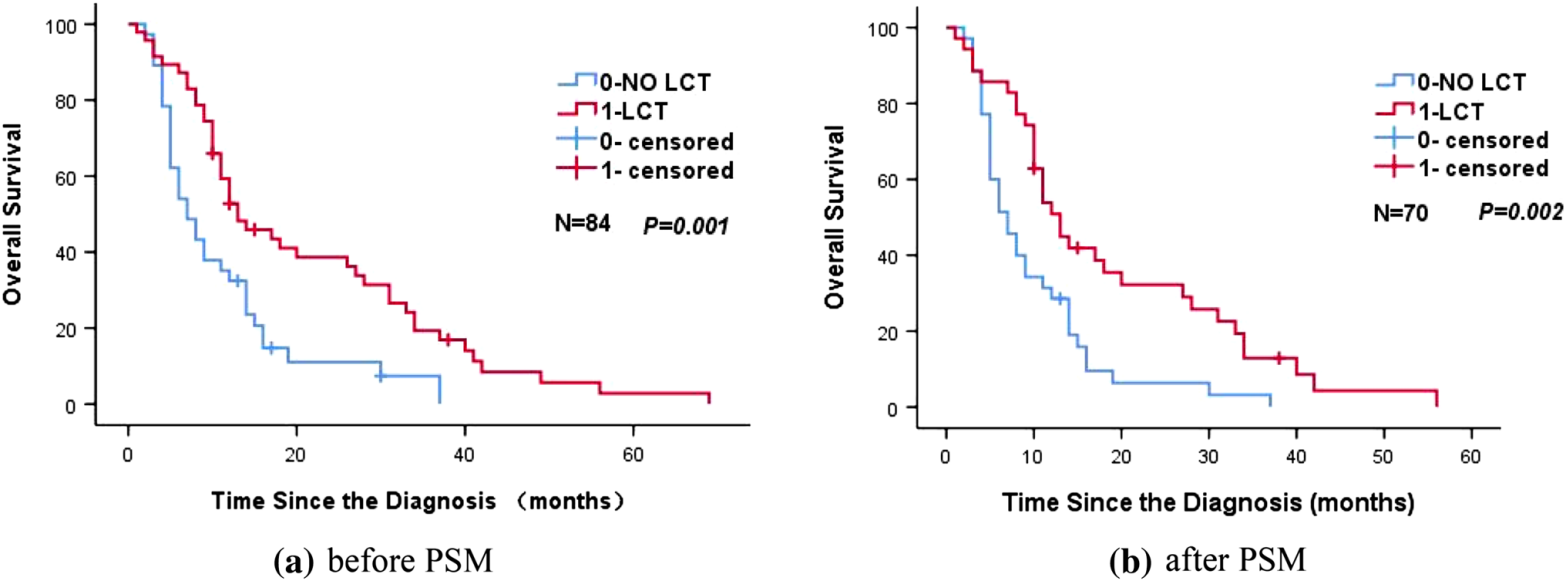
Kaplan–Meier curve of overall survival for patients who did and did not receive LCT

**Fig. 3:**
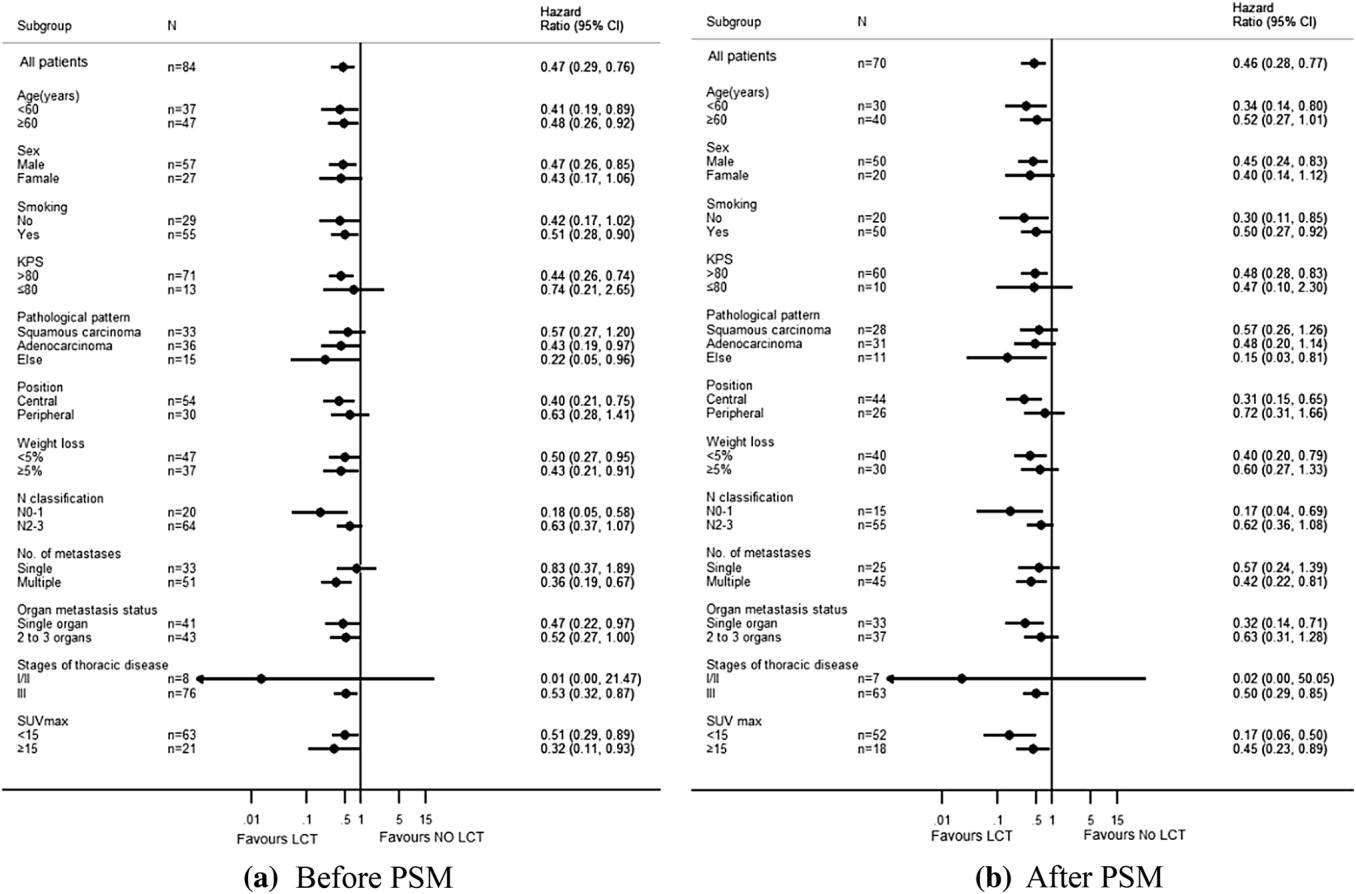
Subgroup analysis of OS among patients: **a** before PSM, **b** after PSM

Tables 2 and 3 show the significant variables from univariate analysis which included LCT status, lymph node metastasis, and weight loss (all *p *< 0.05). Given the heterogeneity of the study sample, we did not evaluate the effects of different chemotherapy regimens on prognosis. Figure 2 shows that, among our patients with oligometastatic stage IV NSCLC, LCT of the tumor site (primary tumor and/or distant metastasis) was associated with a significantly improved median OS (13 months vs. 7 months, *p *= 0.002). Subgroup analyses according to the patients’ clinical characteristics also confirmed that LCT was associated with a significantly longer OS in all subgroups (Fig. 3). Multivariate analysis confirmed that LCT independently predicted a better OS for oligometastatic NSCLC (*p *= 0.001) (Tables 4 and 5).

**Table 2:** Unifactorial analysis of the association between prognostic factors and overall survival (before PSM)

Characteristics	*N*	Median survival time (months)	1-year OS (%)	2-years OS (%)	*X*^2^	*p* value
Age (years)						
< 60	37	13	56.8	27.0	0.421	0.521
≥ 60	47	10	40.4	19.1		
Gender						
Male	57	10	43.9	17.5	0.445	0.505
Female	27	14	55.6	33.3		
Smoking						
No	29	12	48.3	24.1	1.167	0.28
Yes	55	10	47.3	21.8		
KPS						
> 80	71	11	45.1	22.5	0.465	0.495
≤ 80	13	14	61.5	23.1		
Pathological pattern						
SQC	33	10	42.4	15.2	1.318	0.517
AD	36	11	47.2	25.0		
Other	15	14	60.0	33.3		
Position						
Central	54	11	48.1	22.2	0.284	0.594
Peripheral	30	11	46.7	23.3		
Weight loss						
< 5%	47	14	59.6	27.7	0.734	0.392
≥ 5%	37	9	32.4	16.2		
T classification						
T1	1	14	100.0	0.0	0.483	0.923
T2	23	15	47.8	17.4		
T3	29	8	37.9	27.6		
T4	31	12	54.8	22.6		
N classification						
N0–1	20	15	50.0	35.0	4.637	0.031
N2–3	64	11	46.9	18.8		
Number of metastatic sites						
1	32	11	43.8	18.8	2.485	0.647
2	25	9	40.0	20.0		
3	11	17	72.7	27.3		
4	4	5	50.0	25.0		
5	12	8	50.0	33.3		
No. of metastasis						
Single	33	11	42.4	18.2	0.033	0.857
Multiple	51	12	51.0	25.5		
Organ metastasis status						
Single organ	41	11	43.9	22.0	0.258	0.611
2–3 organs	43	12	51.2	23.3		
Stages of thoracic disease						
I/II	8	15	50.0	25.0	2.012	0.156
III	76	11	47.4	22.4		
SUV max						
< 15	63	11	47.6	23.8	0.736	0.391
≥ 15	21	12	47.6	19.0		
Local consolidation therapy						
NO LCT	37	7	35.1	8.1	10.516	0.001
LCT	47	13	57.4	34.0		
Lung metastasis (yes, %)	50	13	58.0	30.0	7.178	0.007
Brain metastasis (yes, %)	5	11	40.0	20.0	0.668	0.414
Bone metastasis (yes, %)	37	11	48.6	21.6	1.102	0.294
Liver metastasis (yes, %)	16	9	31.3	18.8	0.888	0.346
Adrenal metastasis (yes, %)	11	12	54.5	9.1	1.43	0.232
Other metastasis (yes, %)	11	7	36.4	18.2	0.302	0.583
Mixed metastasis (yes, %)	38	11	50.0	23.7	0.192	0.662

*n* number of cases/controls, *PSM* propensity score matching, *LCT* local consolidation therapy, *SQC* squamous cell carcinoma, *AD* adenocarcinoma, *Other* other specified carcinoma

**Table 3:** Unifactorial analysis of the association between prognostic factors and overall survival (after PSM)

Characteristics	*n*	Median survival time (months)	1-year OS (%)	2-years OS (%)	*X*^2^	*p* value
Age(years)						
< 60	30	12	53.3	23.3	0.264	0.607
≥ 60	40	10	32.5	12.5		
Gender						
Male	50	9	40.0	16.0	0.029	0.866
Female	20	11	45.0	20.0		
Smoking						
No	20	10	30.0	10.0	1.248	0.264
Yes	50	10	46.0	20.0		
KPS						
> 80	60	10	40.0	18.3	0.018	0.892
≤ 80	10	10	50.0	10.0		
Pathological pattern						
SQC	28	10	39.3	14.3	0.533	0.766
AD	31	11	41.9	16.1		
Other	11	10	45.5	27.3		
Position						
Central	44	9	43.2	15.9	0.031	0.861
Peripheral	26	10	38.5	19.2		
Weight loss						
< 5%	40	13	55.0	25.0	4.912	0.027
≥ 5%	30	8	23.3	6.7		
T classification						
T1	1	14	100.0	0.0	0.123	0.989
T2	20	11	40.0	10.0		
T3	26	8	34.6	23.1		
T4	23	11	47.8	17.4		
N classification						
N0–1	15	15	46.7	26.7	3.654	0.056
N2–3	55	10	40.0	14.5		
Number of metastatic sites						
1	24	11	33.3	12.5	2.028	0.731
2	24	6	37.5	16.7		
3	8	17	62.5	12.5		
4	3	5	33.3	0.0		
5	11	7	54.5	36.4		
No. of metastases						
Single	24	10	33.3	12.5	0.081	0.776
Multiple	46	10	45.7	19.6		
Organ metastasis status						
Single organ	33	10	36.4	15.2	0.096	0.756
2–3 organs	37	11	45.9	18.9		
Stages of thoracic disease						
I/II	7	15	42.9	14.3	0.449	0.503
III	63	10	41.3	17.5		
SUV max						
< 15	52	9	42.3	19.2	0.008	0.93
≥ 15	18	10	38.9	11.1		
Local consolidation therapy						
NO LCT	35	7	31.4	5.7	9.739	0.002
LCT	35	13	51.4	28.6		
Lung metastasis (yes, %)^d^	41	12	51.2	24.4	4.853	0.028
Brain metastasis (yes, %)	3	5	0.0	0.0	2.388	0.122
Bone metastasis (yes, %)	33	10	45.5	18.2	1.169	0.28
Liver metastasis (yes, %)	15	9	26.7	13.3	0.221	0.639
Adrenal metastasis (yes, %)	8	12	62.5	12.5	0.046	0.83
Other metastasis (yes, %)	9	6	22.2	0.0	1.817	0.178
Mixed metastasis (yes, %)	33	10	45.5	18.2	0.224	0.636

*n* number of cases/controls, *PSM* propensity score matching, *LCT* local consolidation therapy, *SQC* squamous cell carcinoma, *AD* adenocarcinoma, *Other* other specified carcinoma

**Table 4:** Multivariate analysis between prognostic factors and overall survival (before PSM)

Factor	*B*	Sx	*p*	HR (95% CI)
N classification (N0–1 vs. N2–3)	0.493	0.311	0.113	1.637 (0.89–3.011)
Local consolidation therapy (no LCT vs. LCT)	− 0.678	0.250	0.007	0.508 (0.311–0.828)

**Table 5:** Multivariate analysis between prognostic factors and overall survival (after PSM)

Factor	*B*	Sx	*p*	HR (95% CI)
N classification (N0–1 vs. N2–3)	0.562	0.347	0.105	1.755 (0.888–3.467)
Local consolidation therapy (no LCT vs. LCT)	− 0.999	0.291	0.001	0.368 (0.208–0.651)

## Discussion

Most deaths caused by NSCLC are related to the development and growth of distant metastases, which highlights the importance of systemic disease control (Fidler 2003). However, optimal treatment selection remains challenging for this patient group. A large trial investigated the potential benefit of four platinum-based doublet chemotherapy regimens (cisplatin plus either paclitaxel, gemcitabine, or docetaxel, and carboplatin plus paclitaxel) in 1155 patients with metastatic NSCLC; however, the results revealed that none of these regimens were significantly superior (Schiller et al. 2002). This finding may be related to the variable biology of metastatic lesions, which might respond differently to therapy (vs. the primary tumor), and highlights the importance of biological characterization if metastatic lesions. Pretreatment tumor characterization using PET might help guide treatment selection, such as surgery, RT, or specific chemotherapy regimens. Although it seems intuitive that improving patient selection and tumor targeting would improve the results of RT, there is little information regarding long-term patient outcomes to justify the use of PET/CT for planning LCT in cases of oligometastatic stage IV NSCLC. Our earlier study on FDG-PET scanning (Wang et al. 2012a) revealed that this technique influenced patient selection for curative treatment and frequently changed the RT target volumes. The present study investigated PET/CT-based management using different regimens (chemotherapy vs. chemotherapy plus LCT), which revealed that chemotherapy plus LCT was associated with better long-term survival. Furthermore, our treatment results tended to be better than previously reported results for stage IV NSCLC (Song et al. 2018). We suspect that the PET/CT evaluation might have improved the selection of surgery or RT for patients with NSCLC and previously unknown metastatic disease. The PET/CT evaluation may also increase the likelihood of correctly delineating the tumor tissue. For example, we have previously reported (Wang et al. 2012b) that using FDG-PET during RT planning for NSCLC helped improve the reliability of target volume delineation and allowed for a higher tumor dose without an increased risk of side effects. Thus, by more reliably confining the planning target volume to morphologically and functionally visible tumor manifestations, less normal tissue would be irradiated, and a higher total tumor dose would be possible. Several studies have shown that PET/CT planning for NSCLC patients allowed for a significantly higher threshold for the intolerable total dose (68.9 Gy vs. 55.2 Gy for CT alone), on the basis of commonly accepted dose restrictions for normal tissues (De Ruysscher et al. 2005; Greco et al. 2007; Gregoire et al. 2007; Grills et al. 2007; MacManus et al. 2009; Nestle et al. 2002).

Oligometastatic NSCLC is a unique disease and research has focused on improving outcomes using appropriate management strategies. Several retrospective studies (Gomez et al. 2016; Iyengar et al. 2014; Petty et al. 2018) have suggested that LCT has a role in the management of oligometastatic NSCLC, with selection criteria involving lymph node status, tumor histology, thoracic disease bulk, performance status, and number of metastatic sites. On the basis of these criteria, LCT may help improve the effects of systemic treatment for lung cancers. For example, Iyengar et al. (Iyengar et al. 2014) evaluated 24 patients with advanced NSCLC who experienced progression after platinum-based chemotherapy and reported that 16 patients underwent stereotactic body radiation therapy. The results indicated that RT provided significantly better PFS and OS, relative to historical values for patients who had received only systemic treatment. A later prospective study (Iyengar et al. 2018) involved 29 patients with oligometastatic stage IV NSCLC, including 14 patients who were treated using stereotactic ablative radiotherapy (SAbR) plus maintenance chemotherapy and 15 patients who were treated using maintenance chemotherapy. That trial was closed early after an interim analysis revealed that SAbR plus maintenance chemotherapy yielded significantly improved PFS (9.7 months vs. 3.5 months, *p *= 0.01). In addition to the nearly tripling of the PFS value in that trial, the use of consolidative SAbR before maintenance chemotherapy did not increase the risk of toxicities in patients with limited metastatic NSCLC. Petty et al. (2018) also reported on 27 patients who fulfilled the criteria for combined RT. Although the study was ended prematurely because of slow patient accumulation, the results fulfilled the primary endpoint for success (PFS > 6 months, *p *< 0.0001), with a median PFS of 11.2 months (95% CI 7.6–15.9 months) and a median OS of 28.4 months (95% CI 14.5–45.8 months). Thus, among patients with oligometastatic NSCLC, intensive RT combined with non-maintenance chemotherapy after chemotherapy appears to yield significant long-term efficacy. Further studies are needed to validate the efficacy of LCT and standardize its use.

Gomez et al. (2016) conducted the first clinical trial of LCT plus standard maintenance therapy for all sites, which revealed a median PFS of 11.9 months (90% CI 5.7–20.9 months) in the LCT group and 3.9 months (90% CI 2.3–6.6 months) in the maintenance group. This difference was statistically significant (HR: 0.35, 90% CI 0.18–0.66; log-rank *p *= 0.0054), and both groups experienced similar side effects, with no treatment-related deaths or grade 4 adverse events. Moreover, the 1-year PFS rates were 48% in the LCT group (90% CI 28.7–65.7%) and 20% in the maintenance treatment group (90% CI 7.1–38.0%). Thus, relative to maintenance therapy/observation, LCT after systemic therapy for oligometastatic NSCLC appears to be feasible and tolerable and to yield a significant improvement in PFS. The researchers also reported that LCT was associated with a significantly longer time to appearance of new lesions. On the basis of these results, LCT appears to have a role in the management of oligometastatic stage IV NSCLC, in addition to traditional systemic therapy, although it is important to note that these data were derived from a few studies in American and French populations (Gomez et al. 2019; Rusthoven et al. 2009; Su et al. 2016; Zhang et al. 2018). Given the regional and ethnic differences in NSCLC cases, further studies are needed to confirm that LCT does indeed have a good efficacy, with acceptable side effects, in other populations. To the best of our knowledge, ours is the first study to address this issue in a cohort of Chinese patients with oligometastatic NSCLC who have unique genotypic and clinical characteristics. All of the patients in the present study received systemic chemotherapy to control their disease, and our results suggest that LCT may help address any lack of chemotherapy efficacy without increasing the risk of serious adverse events or treatment-related death. This finding is consistent with the reported results from previous studies. Therefore, despite the limitations related to a small sample size, retrospective design, and heterogeneous patient characteristics, our results suggest that LCT is feasible in this setting and requires further research.

## Conclusions

Stage IV NSCLC is a systemic disease with a very poor prognosis, and the concept of oligometastasis may provide new insights regarding its treatment. For oligometastatic stage IV NSCLC, the combination of active LCT and systemic chemotherapy may help improve survival without increasing the incidence of side effects, relative to maintenance therapy or observation alone. Furthermore, the use of PET/CT may help clearly identify the disease and potentially help improve patient outcomes. However, further studies are needed to develop a standard for selecting LCT in cases of oligometastatic stage IV NSCLC.
